# An Analysis of Optical Coherence Tomography Angiography (OCT-A) Perfusion Density Maps in Patients Treated for Retinal Vein Occlusion with Intravitreal Aflibercept

**DOI:** 10.3390/diagnostics13193100

**Published:** 2023-09-29

**Authors:** Dorota Śpiewak, Katarzyna Witek, Łukasz Drzyzga, Ewa Mrukwa-Kominek

**Affiliations:** 1 Department of Ophthalmology, Prof. K. Gibiński University Clinical Center, Medical University of Silesia, 40-514 Katowice, Poland; 2 Department of Ophthalmology, Faculty of Medical Sciences in Katowice, Medical University of Silesia, 40-055 Katowice, Poland

**Keywords:** central retinal vein occlusion, optical coherence tomography angiography, aflibercept, superficial plexus vessel density, deep plexus vessel density, flow areas in outer retina, choriocapillaris flow area, non-flow area, foveal avascular zone

## Abstract

Aim: The primary goal of this study was to evaluate the reduction in non-perfusion area and improvement in blood flow as well as the reduction in retinal edema on optical coherence tomography angiography (OCT-A) in patients with retinal vein occlusion treated with 2 mg intravitreal injections of aflibercept. Material and methods: Fifty eyes of nine patients with central retinal vein occlusion (CRVO) and sixteen patients with branch retinal vein occlusion (BRVO), aged 50 to 75 years, were collectively analyzed as retinal vein occlusion (RVO). The following parameters were analyzed: superficial vessel density (VDSF), deep vessel density (VDD), flow area in the outer retina (FAOR), choriocapillaris flow area (FACC), non-flow area (NFA) and the foveal avascular zone (FAZ). Results: OCT-A revealed a reduction in macular edema. The most significant change in central retinal thickness (CRT) was observed between measurement timepoint “5” and the baseline (46%). The non-flow area was also reduced. Following a significant decrease in superficial vessel density 30 days after the first dose of aflibercept, a non-significant increase was noted at the subsequent timepoints. An increase was also found in deep vessel density and choriocapillaris flow area. Improvements in the above OCT-A parameters resulted in increased retinal blood flow and improved visual acuity. Conclusions: Patients with retinal vein occlusion treated with 2 mg aflibercept exhibited reduced macular edema and FAZ, increased vessel density, improved blood flow, and better visual acuity.

## 1. Introduction

Retinal vein occlusion (RVO) is the second most common retinal vascular disease after diabetic retinopathy, which can cause irreversible deterioration of visual acuity related to ischemia and resultant macular edema [1,2,3,4,5]. Central retinal vein occlusion (CRVO) and the three times more prevalent branch retinal vein occlusion (BRVO) are conditions that occur most frequently in patients of advanced age and suffering from systemic diseases, such as diabetes, hypertension, hyperlipidemia, and cardiovascular disease. RVO also occurs in some blood diseases due to coagulation and fibrinolysis system imbalances. The unfavorable properties of a fibrin clot, including reduced susceptibility to lysis, occur in patients with RVO and may contribute to the excessive viscosity described in this disease [6,7,8].

Proper treatment of occlusion sequelae is based on timely and accurate diagnosis of the retinal condition. There have been significant changes in the approach to retinal disorders related, among others, to the rapid development of modern diagnostic imaging techniques. These include Optical Coherent Tomography (OCT) and, above all, Optical Coherent Tomography Angiography (OCT-A), a non-invasive imaging modality that visualizes the individual layers of the retina [8]. Despite the introduction of OCT and OCT-A, both fluorescein angiography and indocyanine green angiography remain valuable adjuncts to diagnosing retinal diseases.

Currently, there are several modern therapeutic options for retinal vein occlusion. Vascular endothelial growth factor (anti-VEGF) inhibitors and corticosteroids such as dexamethasone drug delivery system or triamcinolone acetate are commonly used. Systemic preparations, including low-molecular-weight heparin, niacin, and acetylsalicylic acid, have also been administered. Anti-VEGF is currently the standard of care for macular edema secondary to RVO. The efficacy of anti-VEGF has been shown in numerous studies, such as COPERNICUS, GALILEO, and HORIZON [9].

The goal of this study was to evaluate the reduction in the non-perfusion area and improvement in blood flow on optical coherence tomography angiography (OCT-A) as well as the reduction in retinal edema on OCT in patients with thrombotic occlusion of retinal veins treated with 2 mg aflibercept intravitreal injections. In addition, to determine the effectiveness of this treatment based on the examination of retinal vessels, visual field, and visual acuity of the treated and control eyes.

We hypothesized that anti-VEGF treatment in RVO patients would result in detectable changes in central retinal thickness (CRT) and hemodynamic changes within the macula.

## 2. Material and Methods

Fifty eyes of 25 patients with central retinal vein occlusion (CRVO) and branch retinal vein occlusion (BRVO), aged between 50 and 75 years, were included in this study. Based on our own experience and the findings of Balaratnasingam et al. [10], we decided to collectively analyze both conditions as retinal vein occlusion (RVO). There were 25 eyes diagnosed with RVO (study eyes) and 25 healthy fellow eyes (control eyes).

The inclusion criteria were as follows: central retinal vein trunk or branch occlusion of no more than one month duration, cystoid macular edema confirmed on OCT, best corrected visual acuity (BCVA) ≤ 5/10 (0.5) determined on the Snellen chart, intraocular pressure ≤ 21 mmHg, no previous treatment for RVO, OCT-A scan quality index of 8/10 or higher, age 50 to 75 years, written informed consent for drug administration (2 mg aflibercept) and venous blood collection for testing.

Exclusion criteria included hypersensitivity to the drug, advanced glaucoma resistant to pharmacotherapy, infection of the eye or its area, vitreous hemorrhage, vitreoretinal traction, ocular media opacity affecting the quality of OCT-A scans), age below 50 years or above 75 years, systemic bacterial, viral or parasitic infection, and lack of written informed consent.

Intravitreal injections of 2 mg aflibercept were given three times at 30-day intervals.

Follow-up examinations were conducted 30 days after each intravitreal injection of anti-VEGF and at monthly intervals for two months after the last aflibercept injection. A total of six measurements were made, including

- pre-therapy measurement, i.e., timepoint 0 (hereafter referred to as the baseline);
- three intra-therapy timepoints, i.e., timepoint 1 (30 days after the first anti-VEGF injection), timepoint 2 (30 days after the second injection), and timepoint 3 (30 days after the third injection);
- two post-therapy timepoints, i.e., timepoint 4 (60 days after the third injection) and timepoint 5 (90 days after the third injection).

Descriptive and analytical statistics were used to analyze these data. Descriptive statistics show the distributions of individual variables. The mean value, standard deviation, middle value (median), and value range (minimum-maximum) were calculated for quantitative variables. The normality of the distribution of quantitative variables was tested using the Shapiro–Wilk test; the level of significance was set at *p* < 0.05. The determination of qualitative variables distribution included calculating their individual values.

Analytical statistics allowed the determination of the statistical significance of differences. Differences between the distributions of quantitative variables in two independent groups were assessed. A Student’s *t*-test was used when the distribution of variables did not deviate from a normal distribution and the non-parametric Kruskal–Wallis test when the distribution of variables was non-normal. A paired samples *t*-test was used to assess paired differences when the distribution of variables was normal, and the non-parametric Wilcoxon Signed Rank test was used when the distribution of variables was other than normal. Data analysis was based on variables reflecting the measured values and transformed variables.

A series of measurements taken at the following timepoints were analyzed: the pre-therapy timepoint (point 0) and five monitoring points at designated intervals (points 1–5).

The dynamics of changes in the results of the ophthalmic examination were evaluated by analyzing the absolute values and transformed variables. Variables were transformed using standardization of repeated measurement results against the initial measurement (initial measurement value = 100%). Relative values (%) of changes in the study parameters were calculated for each measurement timepoint (point 0 vs. point 1, point 0 vs. point 2, point 0 vs. point 3, point 0 vs. point 4, point 0 vs. point 5). The results of difference and correlation analyses were interpreted using the statistical significance level of *p* < 0.05.

### 2.1. Acquisition of Images

OCT and OCT-A were performed using the Angiovue optical coherence tomography angiography (OCTA) software of the RTVue XR Avanti (Version 2018.1.1.69, Optovue Inc., Fremont, CA, USA).

### 2.2. OCTA Analysis

OCT-A images of the 6 × 6 mm central macular area were used for analysis. The following parameters were analyzed:

- vessel density (VD) of the superficial capillary plexus (VD-SF) and deep capillary plexus (VD-D),
- flow area in the outer retina (FAOR) and choriocapillaris flow area (FACC),
- superficial non-flow area (NFA),
- foveal avascular zone (FAZ).

Central retinal thickness (CRT) was determined based on OCT images and ETDRS grids.

Automated measurement was used for OCT and OCT-A parameters, except for NFA, which was measured manually by one examiner.

**Figure 1:**
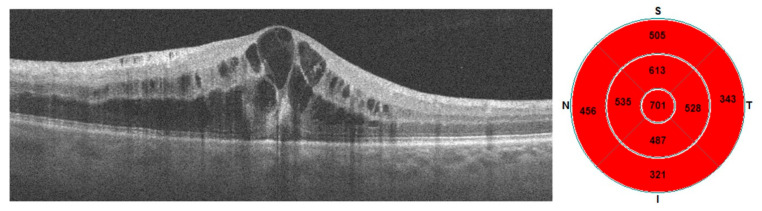
Pre-treatment OCT and ETDRS grid.

**Figure 2:**
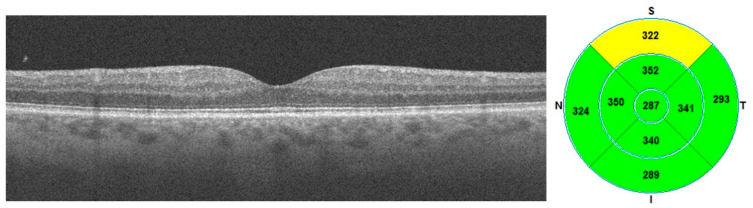
Post-treatment OCT and ETDRS grid.

Below are samples of macular OCT images with the macular thickness map—ETDRS grids before and after the treatment (Figure 1 and Figure 2).

## 3. Results

Standard care for retinal vein occlusion with secondary cystoid macular edema consists of intravitreal administration of a VEGF inhibitor [11].

### 3.1. Intravitreal Anti-VEGF (Aflibercept) and CRT

The analysis of the relative differences at five follow-up measurement timepoints showed a statistically significant decrease in CRT in the eyes with RVO treated with an anti-VEGF (aflibercept). The most remarkable relative CRT decrease (37%) was noted at measurement timepoint 1, i.e., 30 days after the first anti-VEGF injection. These changes were less pronounced at subsequent timepoints but still significant compared with the baseline. The comparison with standardized CRT values was intended to enrich these currently available data with some insight into the dynamics of the processes under study.

**Figure 3:**
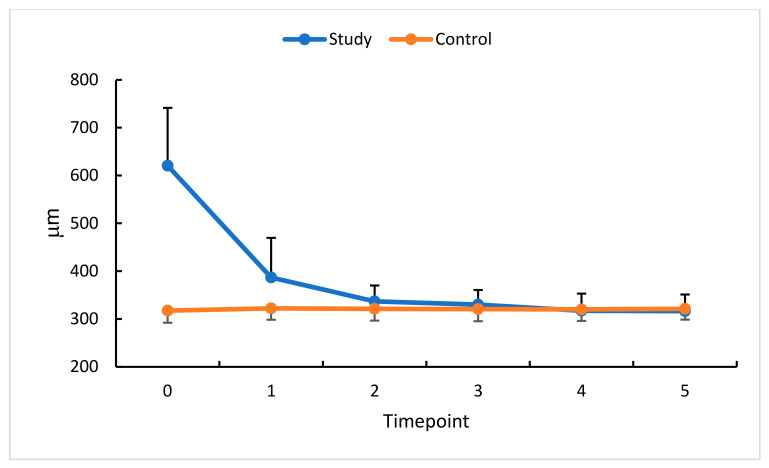
CRT changes (μm) in the study (S) and control (C) eyes; measurement timepoint 0 (the baseline) and subsequent measurements (1 to 5). Color dots represent the mean, and the bars represent the standard deviation.

The most significant decrease in CRT was observed between timepoint 5 and the baseline (46%). The change in CRT values between timepoints 5 and 3 was the lowest (4%). The therapeutic effect (i.e., decrease in CRT) continued until measurement timepoint 3 (30 days after the third anti-VEGF injection). This was followed by stabilization until measurement timepoint 5, i.e., 90 days after the last dose of aflibercept (Figure 3). In the control group, no significant CRT changes were revealed in the time intervals studied.

### 3.2. Intravitreal Anti-VEGF (Aflibercept) and NFA

**Figure 4:**
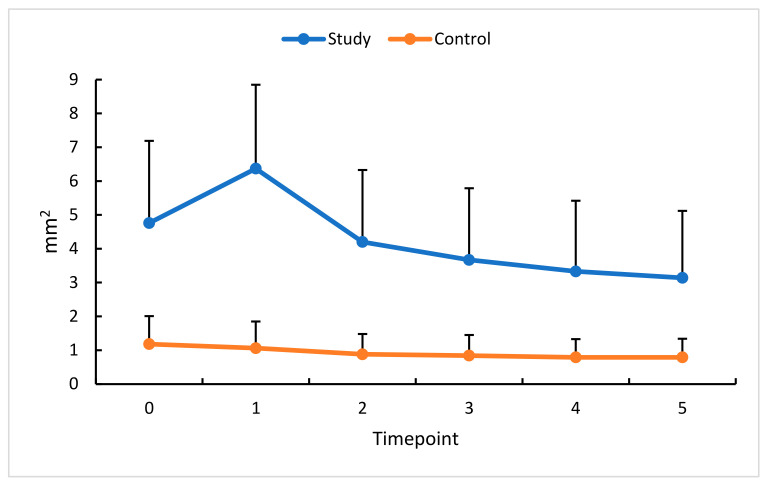
NFA changes (mm^2^) in the study (S) and control (C) eyes; measurement timepoint 0 (the baseline) and subsequent measurements (1 to 5). Color dots represent the mean, and the bars represent the standard deviation.

Thirty days after the first anti-VEGF injection (measurement timepoint 1), a 31% increase in NFA was observed in the treated eye, followed by a decrease at all subsequent measurement timepoints. This might be accounted for by the high likelihood of baseline measurement inaccuracy due to increased congestion of the examined area, retinal edema, and venous dilatation partially masking the NFA surface in this phase of the disease. With the therapeutic effect of anti-VEGF treatment, the NFA decreased significantly (Figure 4).

The mean NFA change in this study’s eyes reached 30%.

### 3.3. Intravitreal Anti-VEGF (Aflibercept) and VD

Another parameter studied was vessel density (VD) in the superficial and deep plexuses. OCT-A showed VD reduction in the study eyes compared with the control.

An analysis of this study’s eyes absolute and relative VDSF values confirmed a significant reduction in this parameter at timepoint 1. A non-significant increase was found at timepoints 2 through 4. Ultimately, i.e., at timepoint 5 (90 days after the third anti-VEGF injection), VDSF returned to the baseline value.

**Figure 5:**
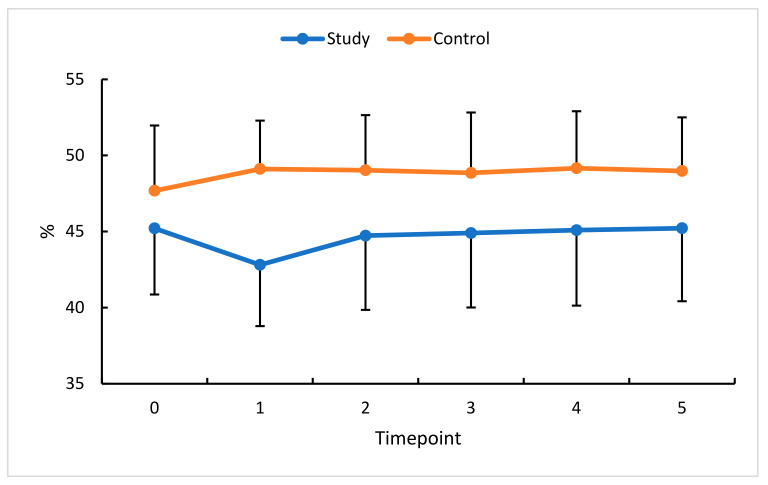
VDSF changes (%) in the study (S) and control (C) eyes; measurement timepoint 0 (the baseline) and subsequent measurements (1 to 5). Color dots represent the mean, and the bars represent the standard deviation.

**Figure 6:**
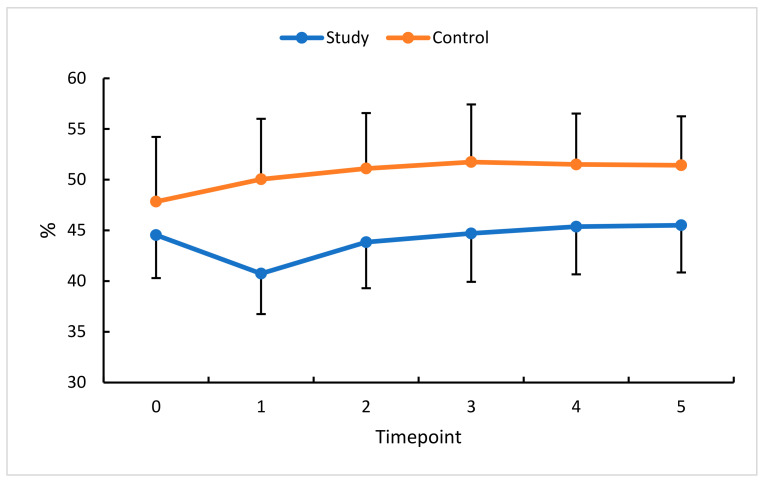
VDD changes (%) in the study (S) and control (C) eyes; measurement timepoint 0 (the baseline) and subsequent measurements (1 to 5). Color dots represent the mean, and the bars represent the standard deviation.

In this study’s eyes, a statistically significant reduction in VDD was only observed between the baseline and timepoint 1. The values obtained at subsequent measurements did not differ significantly compared with the baseline. In the control group, statistical significance was only found at timepoint 1. Statistical analysis showed no statistically significant differences between pre- and post-therapy VDSF or VDD, indicating anti-VEGF therapy for RVO did not significantly affect this parameter. However, observation of the retina after the first injection, when its swelling becomes reduced, and the actual vascularization of the retina is revealed, showed an increasing trend at timepoints 2 through 5 (Figure 5 and Figure 6).

An analysis of the percentage change in VDSF revealed little change (0% to 1% in this study’s eyes and 0% to 3% in the control eyes), indicating the insensitive nature of this parameter for assessing and documenting the effects of therapy. Changes in VDD were delayed beyond the therapy phase but then persisted until the end of the observation period (i.e., until timepoint 5). Notably, in the control eyes, small changes in VDSF and VDD were seen at timepoint 1 and persisted until the end of the follow-up period.

To extend our analysis of RVO-related changes in retinal hemodynamics, the flow area in the outer retina (FAOR) and choriocapillaris flow area (FACC) were also assessed.

### 3.4. Intravitreal Anti-VEGF (Aflibercept) and FAOR/FACC

An analysis of FACC change in this study group revealed a statistically significant increase at all measurement timepoints compared with the baseline. Comparisons with standardized data show a substantial increase in FACC at timepoints 1 and 2, each by 10%.

The profiles of change in absolute and relative FAOR values in the study and control groups demonstrate a statistically significant reduction in this study group for all measurement timepoints relative to the baseline. The most remarkable reduction in FAOR values (23%) was observed 30 days after the first anti-VEGF injection, i.e., at timepoint 1. Following a decline in retinal congestion, an increase in perfusion was revealed at all subsequent timepoints, documenting the effectiveness of the therapy.

**Figure 7:**
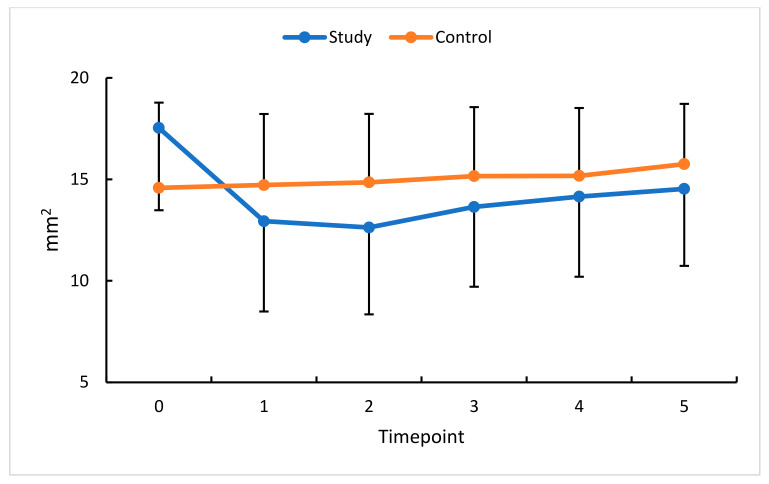
FAOR changes (mm^2^) in the study (S) and control (C) eyes; measurement timepoint 0 (the baseline) and subsequent measurements (1–5). Color dots represent the mean, and the bars represent the standard deviation.

**Figure 8:**
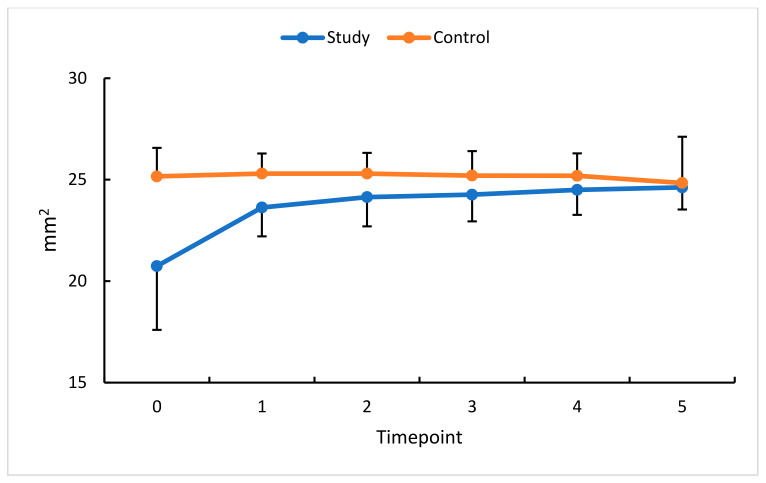
FACC changes (mm^2^) in the study (S) and control (C) eyes; measurement timepoint 0 (the baseline) and subsequent measurements (1–5). Color dots represent the mean, and the bars represent the standard deviation.

An analysis of FAOR change in this study’s eyes revealed a non-unidirectional trend. The change in FACC values was the lowest between timepoints 5 and 3. The greatest therapeutic effect of anti-VEGF was already apparent at measurement timepoint 3 and persisted until timepoint 5 (Figure 7 and Figure 8). In the control group, no significant changes were obtained in the time intervals studied.

### 3.5. Intravitreal Anti-VEGF (Aflibercept) and FAZ

An interesting and important OCT-A parameter to assess the severity of hemodynamic changes in RVO is the foveal avascular zone (FAZ). The profile of FAZ change in this study group showed a gradual and statistically significant increase at successive measurement timepoints compared with the baseline. FAZ values were higher in the treated than in the control eye except for the baseline and timepoint 1. This might be accounted for by the likelihood of OCT-A measurement inaccuracy due to macular edema still present at these two timepoints. No significant differences between successive measurements were noted in the control group.

**Figure 9:**
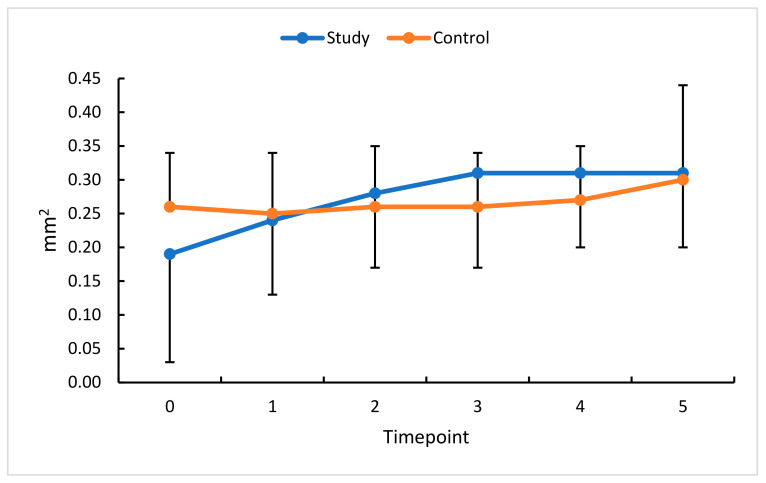
FAZ changes (mm^2^) in the study (S) and control (C) eyes; measurement timepoint 0 (the baseline) and subsequent measurements (1–5). Color dots represent the mean, and the bars represent the standard deviation.

The most remarkable change in FAZ was seen between timepoint 3 and the baseline. The percentage change between timepoints 5 and 3 was the lowest. The most noticeable therapeutic effect of anti-VEGF was already apparent at measurement timepoint 3 and persisted until timepoint 5 (Figure 9). In the control group, no significant changes were obtained in the time intervals studied.

### 3.6. Intravitreal Anti-VEGF (Aflibercept) and BCVA

**Figure 10:**
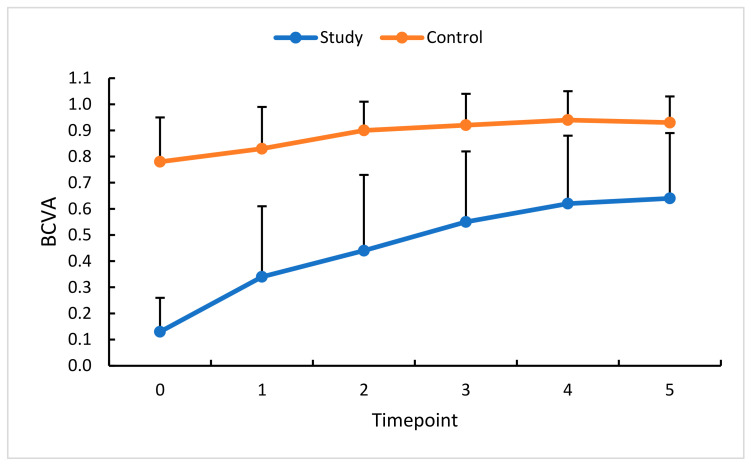
BCVA at the baseline and subsequent measurement timepoints. Color dots represent the mean, and the bars represent the standard deviation.

Best Corrected Visual Acuity (BCVA) is the clinical result of all the hemodynamic variables described above. In this study’s eyes, significantly higher BCVA values were observed throughout the observation period compared with the baseline. Significant differences were also noted for the control eyes. We therefore conclude that the anti-VEGF therapy improved BCVA (Figure 10).

### 3.7. Intravitreal Anti-VEGF (Aflibercept) and MD

**Figure 11:**
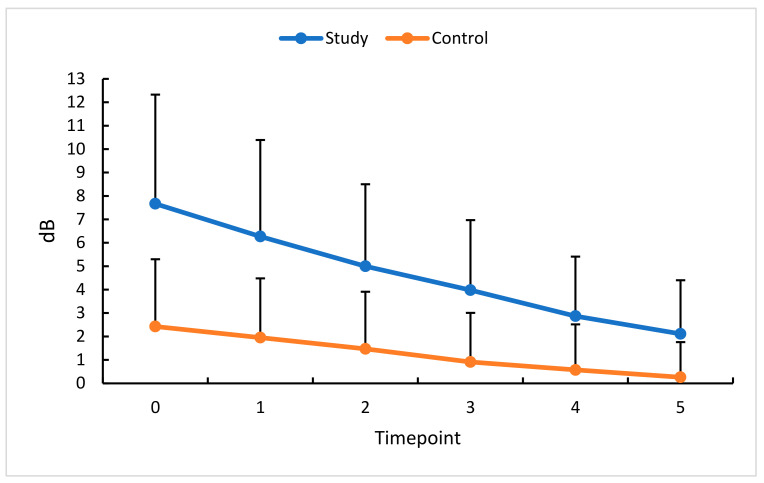
MD changes (dB) in the study (S) and control (C) eyes; measurement timepoint 0 (the baseline) and subsequent measurements (1–5). Color dots represent the mean, and the bars represent the standard deviation.

The last parameter evaluated in this study was the visual field expressed as the mean defect (MD). The study group’s absolute and relative MD values were significantly reduced at successive measurement timepoints, indicating visual field improvement. Similar changes were found in the fellow, i.e., control eyes. Visual field testing performed after consecutive anti-VEGF injections made us conclude that three injections over 90 days stabilized the visual field area in patients with RVO. It should be emphasized that, in the case of other study parameters, sustained response and stability of treatment results were observed after the last aflibercept administration. MD, on the other hand, continued to decrease, indicating further improvement in the visual field (Figure 11).

The present study covers a 5-month follow-up period of 25 eyes with RVO, treated with a monthly intravitreal injection of 2 mg aflibercept (three injections were administered to each eye), and of the same number of fellow eyes of the same patients. The therapy was found to be highly effective.

Our results indicate that the effectiveness of anti-VEGF therapies is most accurately measured with CRT, FAOR, FACC, and FAZ. It should also be noted CRT, FACC, and FAZ showed the highest differentiating ability (a statistically significant difference between the mean change and post-therapy change—diseased eye vs. fellow eye). The analysis of the 2-month post-therapy follow-up revealed a statistically significant difference between post-therapy changes in CRT, NFA, VDD, FACC, and MD for the diseased vs. fellow eye. An important practical observation indirectly derived from this study is that measurements assessing hemodynamic changes in the retina should be taken after retinal edema has resolved.

The presented study had several limitations, most notably a small number of study eyes and the lack of a suitable control group, i.e., untreated RVO patients. Other significant limitations of this study were a short follow-up period and the small number of therapeutic interventions, i.e., only three intravitreal injections. However, despite these drawbacks, we demonstrated some therapy effects. In addition, the changes observed during therapy are consistent with other authors’ observations from analogous clinical trials.

The pathomechanism of retinal vein occlusion and its diagnosis and treatment are still controversial. The increasing number of these patients, new diagnostic techniques, and therapeutic approaches are raising the ophthalmologists’ and patients’ expectations. Further research into, among others, the size of the perfusion zones and the topography of the non-perfusion field, depending on the type and stage of therapy for retinal vein occlusion, is therefore well warranted.

## 4. Discussion

An analysis of relative differences at measurement points 1 through 5 showed a statistically significant decrease in CRT in treated eyes. The therapeutic efficacy of aflibercept was apparent at timepoint 2 and persisted throughout the follow-up period. Huang et al. [12] and Suzuki et al. [13] evaluated CRT in a slightly different protocol but showed a reduction in this parameter following anti-VEGF therapy. Nicolai et al. [14] documented CRT reduction after the first intravitreal injection; this effect persisted four months after the drug had been administered.

In this study group, a significant reduction was found in the NFA’s absolute and relative values starting at timepoint 2. Subsequent anti-VEGF injections caused a reduction in the non-perfusion area and an improvement in regional blood flow. Similar results were obtained by Suzuki et al. [13], who assessed non-perfused areas of the retinal superficial and deep capillary layers. They also found that a higher number of monthly intravitreal injections enhanced the reduction in the NFA area. Post-anti-VEGF reduction in the non-perfusion area in the superficial capillary plexus and deep capillary plexus, with a greater decrease in the deep capillary plexus, was also mentioned by Tsai et al. [15]. They confirmed the effect was more pronounced in eyes that had received more injections and that vessel density did not change after a single anti-VEGF administration. They also suggested that more frequent inhibition of VEGF receptors might induce reperfusion of NFAs and improve blood flow. A detailed analysis of fluorescein angiograms in the BRAVO and CRUISE trials has shown that patients who did not receive anti-VEGF treatment exhibited progression of retinal non-perfusion compared with those with VEGF blockade. The conclusion was that timely administration of an intravitreal anti-VEGF (ranibizumab) may prevent the worsening of retinal non-perfusion and promote reperfusion [16]. The effect of anti-VEGF therapy on the NFA size is still unclear. Some studies have shown NFA reduction and improvement in retinal flow [13,16], while others obtained contradictory results [17,18]. Huang et al. [12] found the final NFA was significantly increased, but a detailed analysis showed NFA decreased in the first and second months only to rise again in the following months of therapy. These results suggest that although retinal vascular flow initially improved, the collateral circulation was not restored. In a study by Choi et al. [19], large non-perfusion areas strongly correlated with macular edema recurrence.

Non-invasive microcirculation imaging with OCT-A showed a reduction in retinal vessel density in the superficial and deep plexuses of RVO eyes compared with the control.

VDSF and VDD showed no statistically significant difference during the entire follow-up period and returned to the baseline at the last measurement timepoint. However, the observation of the retina after the first injection, when edema was reduced, and the actual retinal vascularisation was revealed, showed a favorable trend toward increasing vessel density at subsequent measurement timepoints until the end of this study.

OCT-A images have demonstrated that RVO can be associated with decreases in superficial and/or deep perifoveal vessel density [20,21]. Anti-VEGF therapy reduces the non-perfusion area and improves retinal blood flow, mainly in the deep capillary layer [13,16]. On the other hand, several previous studies have shown that vessel density remains unchanged after anti-VEGF treatment [22,23]. Better perfusion correlates positively with visual acuity after the resolution of edema. Improved visual acuity, in turn, correlates strongly with improved retinal perfusion and reduction in retinal ischemia in patients with RVO. Vessel occlusion leads to impaired blood flow, blood turbulence, increased venous pressure, and intraretinal exudates, resulting in vasodilation of the venous and capillary retinal systems.

This might be the reason why the effects of anti-VEGF therapies cannot be conclusively interpreted. Suzuki et al. [13] observed that anti-VEGF treatment had improved retinal blood flow, especially in the deep capillary plexus. On the other hand, DeCarlo et al. [24] and Mane et al. [25] concluded that anti-VEGF therapies did not cause area reperfusion but subsidence of edema and resultant vessel visualization on OCT-A. The reason for these discrepancies is the low quality of OCT-A images resulting from macular edema. Extensive edema affects the accuracy of OCT-A scan assessment due to signal attenuation and difficulty distinguishing retinal plexuses. In addition, changes in the macular microcirculation may also alter the interpretation of OCT-A scans. On the one hand, when extensive macular edema is found, the vascular density may be overestimated due to vasodilatation. On the other hand, if blood flow slows below the detection value (0.3 mm/s) in dilated vessels, the OCT-A apparatus will misread the image as a cystoid surface [26]. It may, therefore, be argued that macular edema masks and/or displaces the vessels, which explains doubts concerning post-edema reperfusion [24,25,27] and justifies the performance of measurements after this phase. Despite the ability to quantify blood flow, the accuracy of OCT-A measurements is also limited by a relatively small field of view, motion artifacts, and vascular segmentation errors, leading to incorrect flow interpretation.

FAZ changes in this study’s eyes at successive measurement points were characterized by a gradual and statistically significant increase compared with the baseline. A comparison of the FAZ in the RVO eyes with that of the fellow eyes during the 5-month follow-up revealed that FAZ values were higher in the diseased eyes. Again, several researchers believe this parameter should only be assessed when macular edema masking the actual area of the FAZ has subsided [12,25,26,28,29,30]. Tripathy et al. [28] did not observe differences in FAZ size between the RVO and unaffected eye before anti-VEGF treatment. This contrasts with the findings of other investigators who mostly showed FAZ enlargement in eyes with RVO [10,26,30]. One reason for this difference is that, in an eye with RVO, FAZ measurement may be affected by macular edema mimicking FAZ enlargement as intraretinal cysts displace the central vascular network. Therefore, Tripathy et al. [28] excluded patients with macular edema secondary to causes other than RVO to avoid segmentation artifacts by intraretinal exudation. Another possible explanation is the selection of patients with more favorable visual function and fixation stability during OCT-A [10,30]. In addition, it cannot be excluded that the lack of significant difference in the initial phase of this study may have been due to spontaneous regression of the disease [31]. During a 10-month observation, Tripathy et al. [28] noted an increase in the FAZ despite the absence of significant changes in perifoveal perfusion. FAZ remained unchanged in healthy eyes. These results are consistent with our findings. Suzuki et al. [13] also found that the FAZ was larger in eyes with RVO compared with untreated fellow eyes. Six months after an intravitreal anti-VEGF therapy, FAZ enlargement was still seen, especially in the superficial capillary plexus [13,26]. FAZ remodeling may suggest dynamic changes in the blood flow in RVO eyes. This is also supported by the findings of other authors [12,13,26]. It should also be noted that, in eyes with fewer injections, the superficial and deep FAZ increased more compared with eyes with more injections. A larger number of anti-VEGF injections may inhibit the enlargement of the FAZ area or delay its enlargement [13]. Results of previously published studies using fluorescein angiography (FA) showed that FAZ was significantly more prominent in the diseased eye than the healthy eye. However, the authors note that the FAZ and its boundaries were obliterated and challenging to identify clearly. Therefore, quantitative analysis of the FAZ with the FA technique is more complex and less accurate; furthermore, a differential analysis of the superficial and deep retinal layers cannot be performed. Visualization and assessment of the FAZ area using OCT-A is more accurate [32]. Samara et al. [26] also used OCT-A and demonstrated FAZ enlargement. The authors of the most recent reports on anti-VEGF therapies revealed no significant differences between diseased and healthy eyes. Sekanazi et al. [33] found significant correlations between the FAZ and peripheral non-perfusion on fluorescein angiography and VD on OCT-A. The authors also concluded that low vascular densities seen on OCT-A might herald retinal neovascularization. The boundary between the FAZ and the adjacent zone of non-perfusion becomes blurred in the eyes with RVO, which signals a worsening of the clinical condition. Other researchers have reported that in eyes with RVO, disruption of the perifoveal capillary network correlates with peripheral retinal ischemia [34].

The BCVA values recorded for the treated eye at each follow-up timepoint were significantly higher than at the baseline. The therapy used in the study protocol resulted in visual acuity improvement. A significant improvement in BCVA occurred solely in the treated eye and was already apparent during treatment. Several studies indicated that visual acuity was strongly related to the FAZ size in the superficial plexus [32], maximum FAZ diameter [35,36], non-perfusion area, and parafoveal vessel density in the deep plexus [20,37], and deep capillary plexus perfusion [13]. The authors mentioned above concluded that FAZ enlargement was associated with decreased visual acuity and indicated that changes in microcirculation observed in RVO patients caused macular edema and resultant visual acuity decrease. In the present study, the final values of CRT and BCVA showed significant treatment-related improvement. A large number of reports have demonstrated a correlation between CRT and BCVA in retinal vein occlusion [12,33,38]. Superficial and deep capillary plexus densities of BRVO and non-BRVO sectors were significantly correlated with the final BCVA for the RVO eyes. More importantly, the deep capillary plexus density of BRVO sectors in both eyes was the most predictive factor for the final visual outcome [12]. Long-term observations of RVO patients revealed relative visual acuity stabilization after consecutive anti-VEGF injections, which coincided with improvements in hemodynamic parameters assessed by OCT-A [39]. Persistent ischemia of the retinal capillaries can cause irreversible damage to the retina and its function, resulting in permanent vision impairment.

In this study group, a statistically significant reduction in the visual field’s absolute and relative MD values was noted at successive measurement timepoints. Similar changes were found in the control eyes. We, therefore, concluded that a total of three intravitreal anti-VEGF injections (administered during three consecutive months) stabilized the visual field in patients with RVO. Similar results were observed by other researchers. Papadia et al. [40] found not only an improvement but also a stabilization of the examined parameters, of which visual acuity, macular thickness, and MD of the visual field improved significantly (*p* < 0.001). Interestingly, changes in all parameters were independent of the number of anti-VEGF injections. MD in the visual field was shown to re-increase, which could be associated with edema recurring between injections.

In the present study, covering a five-month observation period, the effectiveness of the applied therapy was high. The results have shown that the most accurate indicators of therapy effectiveness were CRT, FAOR, FACC, and FAZ. It should be emphasized that CRT, FAZ, and FACC showed the highest differentiating ability, diseased vs. fellow eye.

## 5. Conclusions

Patients with retinal vein occlusion treated with intravitreal injections of 2 mg aflibercept exhibited reduced macular edema and FAZ, increased vessel density, improved blood flow, and better visual acuity.

## 6. Summary of Results in Tables

Table 1, Table 2, Table 3, Table 4, Table 5 and Table 6 below present the tested parameters by OCT, OCT-A, BCVA, and the MD of the visual field in the study and control groups at each timepoint (0–5).

## 7. Examples of OCT-A Scans before and after Treatment

Figure 12, Figure 13, Figure 14, Figure 15, Figure 16, Figure 17, Figure 18, Figure 19, Figure 20, Figure 21, Figure 22 and Figure 23 below present example microvasculature perfusion maps of the retina and the choroid in the OCT-A at timepoint 0 (before treatment) and timepoint 5 (after treatment).

## Figures and Tables

**Figure 12:**
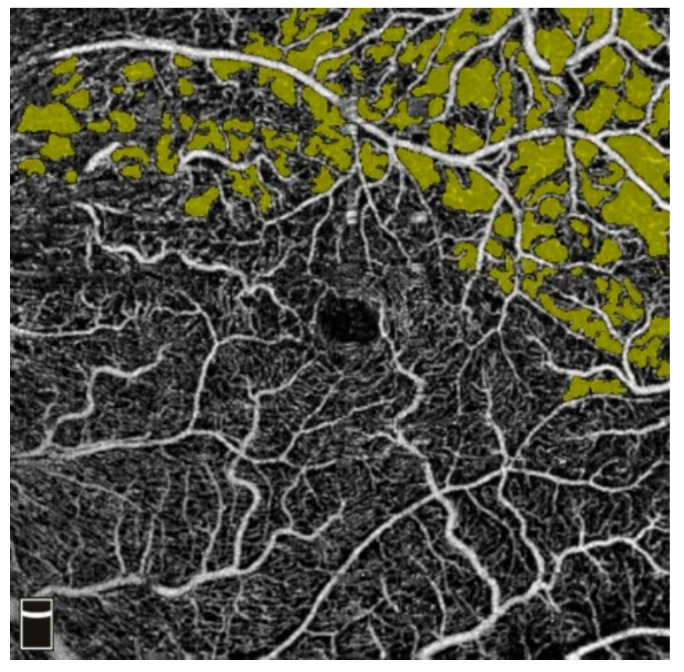
NFA before treatment.

**Figure 13:**
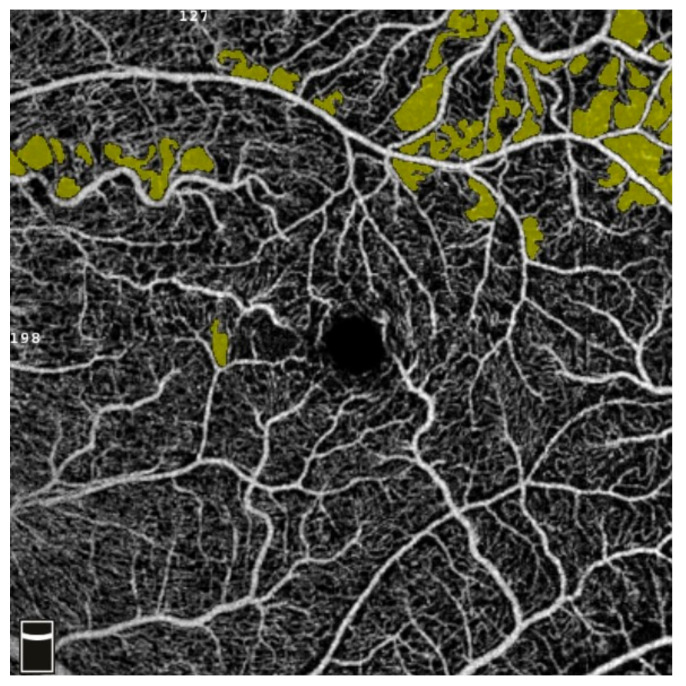
NFA after treatment.

**Figure 14:**
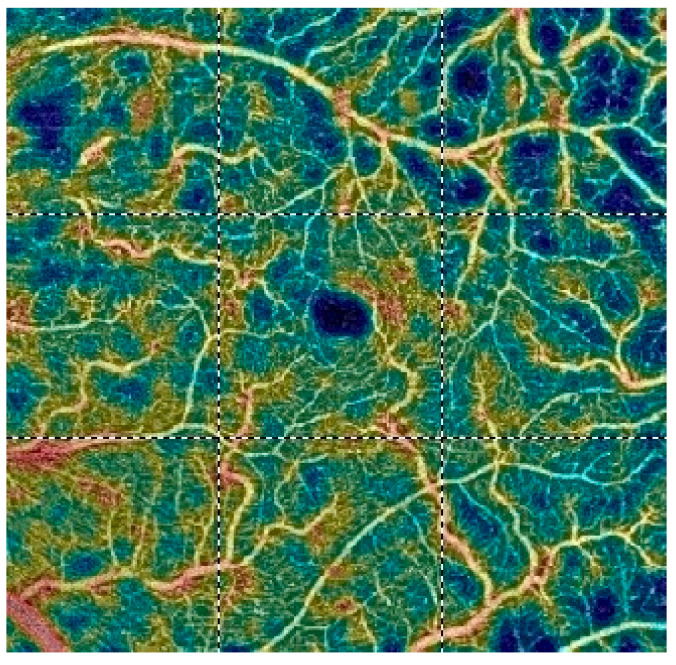
VDSF before treatment.

**Figure 15:**
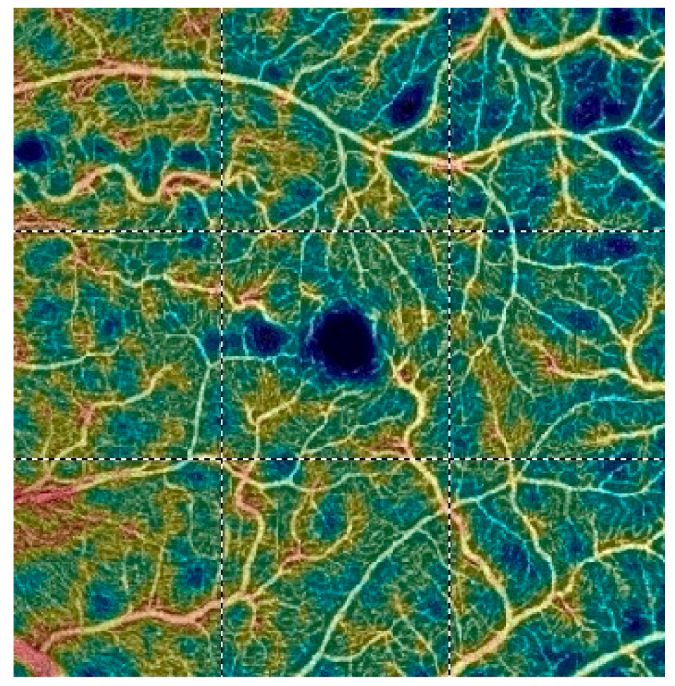
VDSF after treatment.

**Figure 16:**
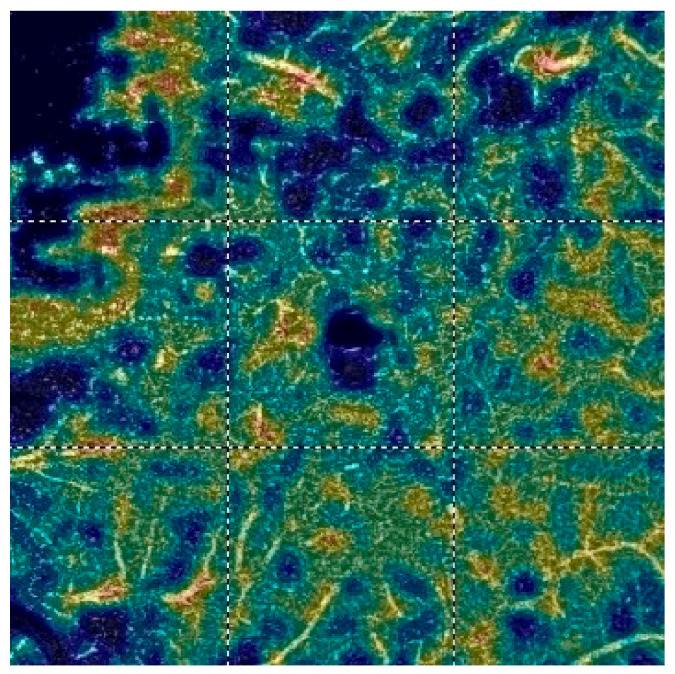
VDD before treatment.

**Figure 17:**
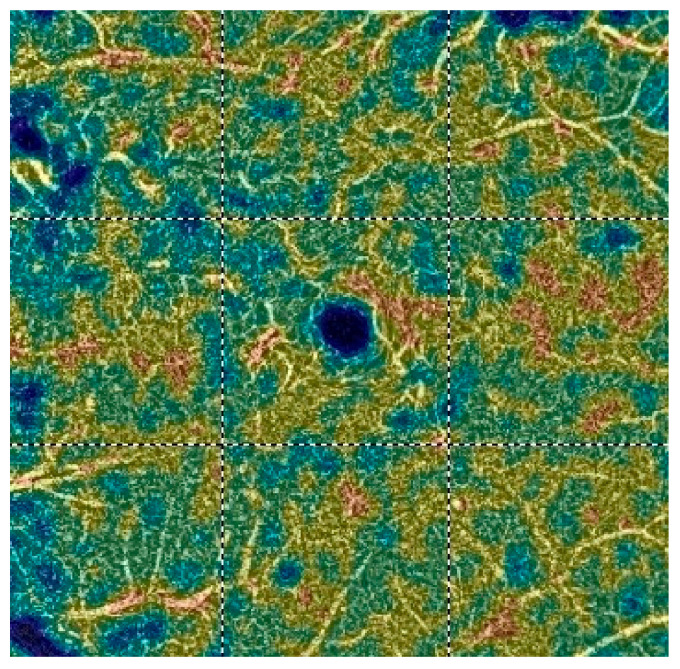
VDD after treatment.

**Figure 18:**
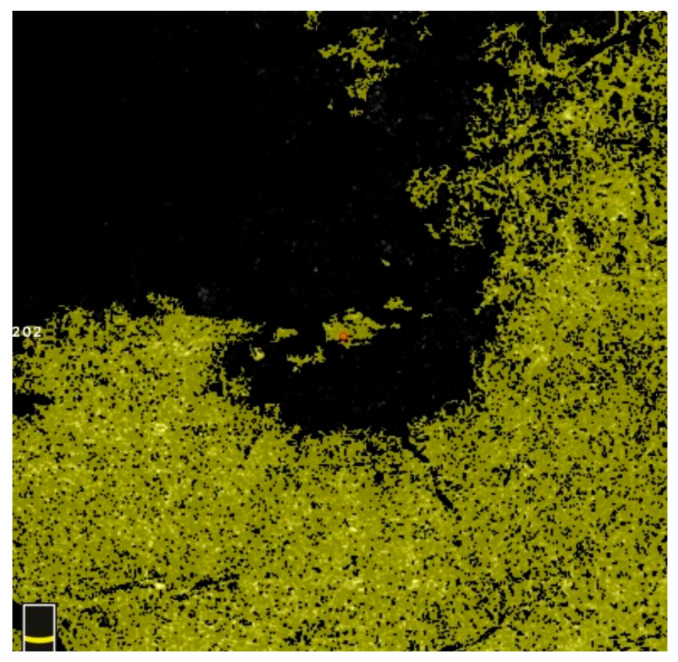
FAOR before treatment.

**Figure 19:**
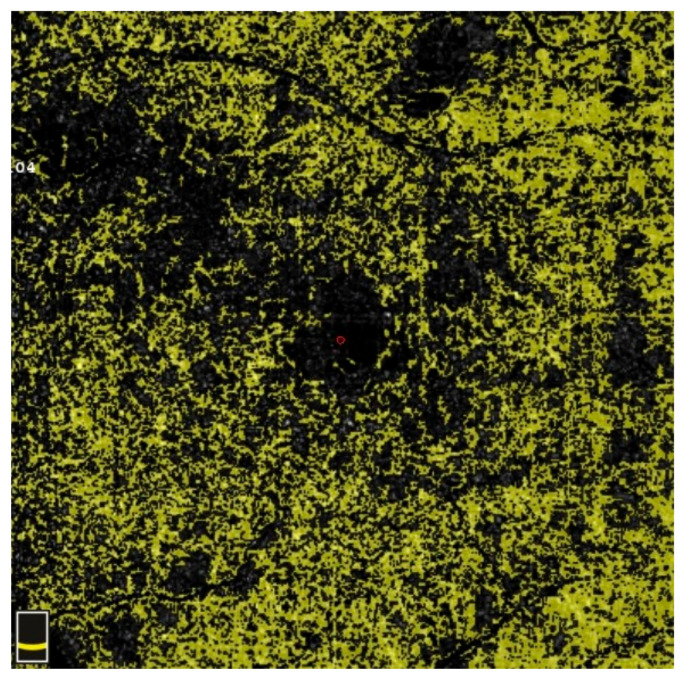
FAOR after treatment.

**Figure 20:**
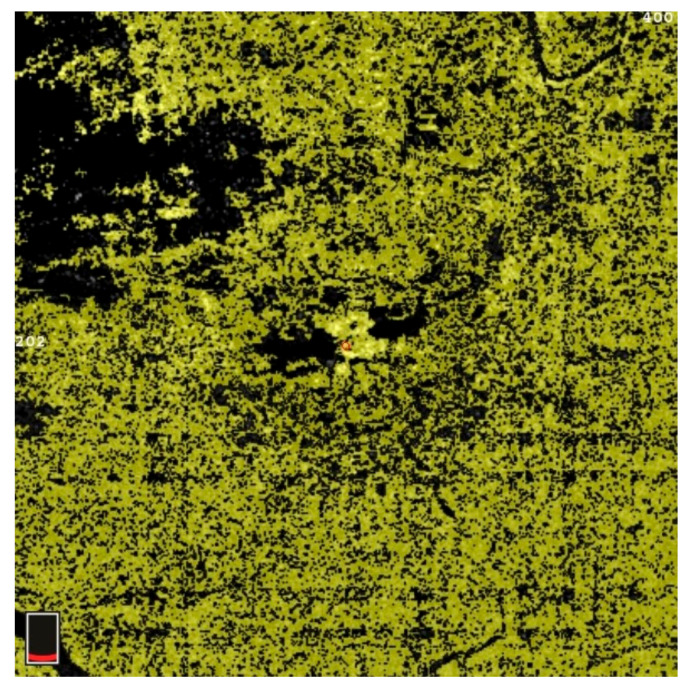
FACC before treatment.

**Figure 21:**
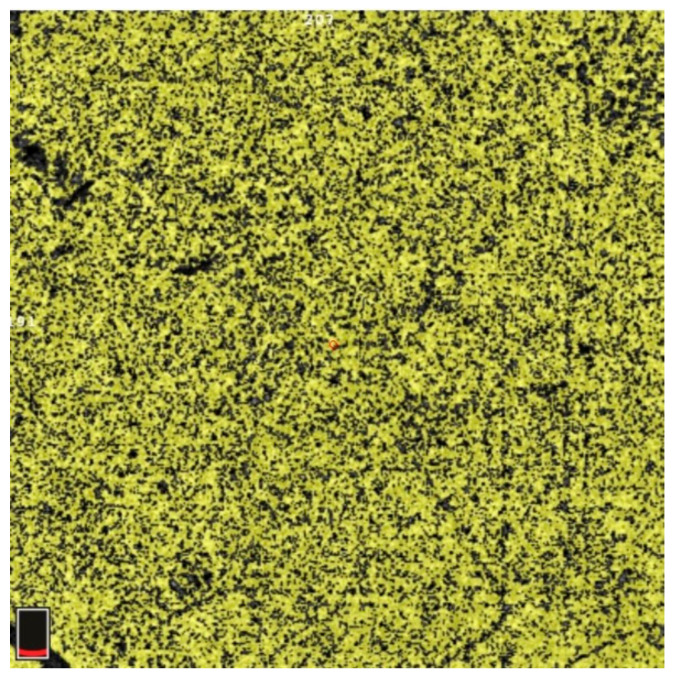
FACC after treatment.

**Figure 22:**
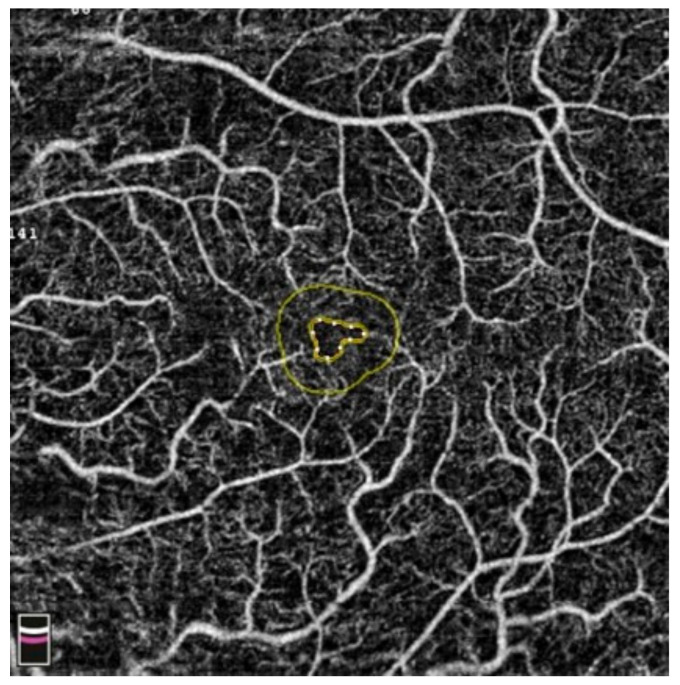
FAZ before treatment.

**Figure 23:**
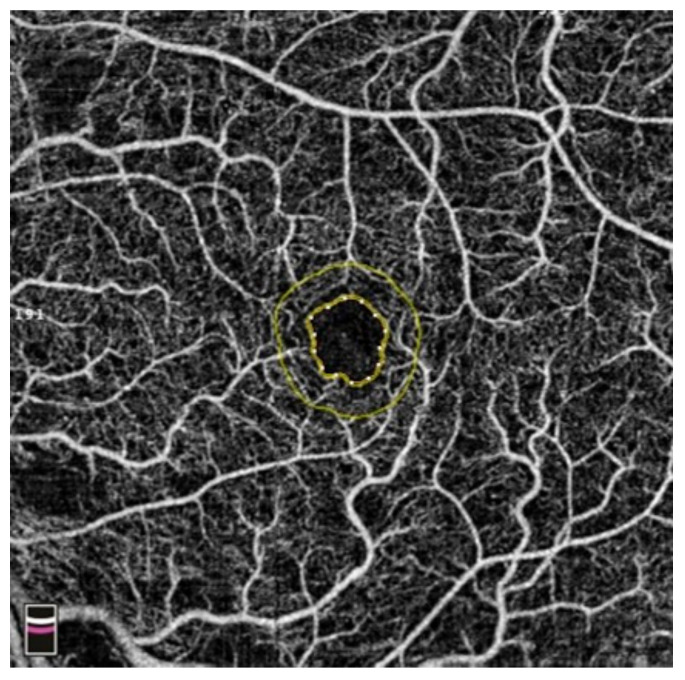
FAZ after treatment.

**Table 1:** Initial evaluation (before injection—timepoint 0)—measurement results in the study (*n* = 25) and control (*n* = 25) eyes and the statistical significance of the difference.

Variable	Study Eyes			Control Eyes			
	X ± SD	Range	Median	X ± SD	Range	Median	*p*-Value
CRT (μm)	620.6 ± 120.9	397–856	632	317.6 ± 25.5	263–384	319	*p* < 0.0001
NFA (mm^2^)	4.76 ± 2.43	1.55–10.66	4.21	1.18 ± 0.83	0.25–3.27	0.95	*p* < 0.0001
VDSF (%)	45.21 ± 4.34	35.2–52.6	44.8	47.68 ± 4.29	35.3–56.0	47.7	*p* = 0.04
VDD (%)	44.54 ± 4.24	36.4–51.3	44.8	47.84 ± 6.38	37.0–59.8	48.8	*p* = 0.03
FAOR (mm^2^)	17.54 ± 4.06	6.7–25.8	17.7	14.58 ± 3.73	8.4–22.3	15.4	*p* = 0.01
FACC (mm^2^)	20.74 ± 3/14	1.70–24.1	21.0	25.16 ± 1.41	21.1–27.2	25.2	*p* < 0.0001
FAZ (mm^2^)	0.19 ± 0.16	0.04–0.92	0.15	0.26 ± 0.08	0.08–0.40	0.27	*p* < 0.001
MD (dB)	7.67 ± 4.66	1.8–17.8	6.3	2.43 ± 2.87	−1.1–9.7	1.3	*p* < 0.0001
BCVA	0.13 ± 0.13	0.02–0.5	0.1	0.78 ± 0.17	0.5–1.0	0.8	*p* < 0.0001

**Table 2:** Timepoint 1—measurement results in the study (*n* = 25) and control (*n* = 25) eyes and the statistical significance of the difference.

Variable	Study Eyes			Control Eyes			
	X ± SD	Range	Median	X ± SD	Range	Median	*p*-Value
CRT (μm)	386.8 ± 82.8	299–653	359	322.2 ± 23.7	272–385	323	*p* < 0.0001
NFA (mm^2^)	6.37 ± 2.48	3.03–12.33	6.15	1.06 ± 0.79	0.15–3.61	0.90	*p* < 0.0001
VDSF (%)	42.81 ± 4.02	34.1–50.2	43.3	49.11 ± 3.18	43.0–57.3	49.0	*p* < 0.0001
VDD (%)	40.74 ± 3.99	35.6–53.6	40.1	50.04 ± 5.97	38.5–62.9	49.3	*p* < 0.0001
FAOR (mm^2^)	12.94 ± 4.45	6.33–21.4	12.8	14.72 ± 3.50	8.5–23.3	14.79	*p* = 0.1
FACC (mm^2^)	23.63 ± 1.42	20.7–26.6	23.6	25.3 ± 0.99	23.3–27.6	25.3	*p* = 0.1
FAZ (mm^2^)	0.24 ± 0.11	0.07–0.55	0.21	0.25 ± 0.08	0.08–0.41	0.24	*p* = 0.3
MD (dB)	6.27 ± 4.12	1.1–15.6	5.5	1.95 ± 2.53	−1.4–8.1	1.1	*p* < 0.0001
BCVA	0.34 ± 0.27	0.04–0.8	0.2	0.83 ± 0.17	0.5–1.0	0.9	*p* < 0.0001

**Table 3:** Timepoint 2—measurement results in the study (*n* = 25) and control (*n* = 25) eyes and the statistical significance of the difference.

Variable	Study Eyes			Control Eyes			
	X ± SD	Range	Median	X ± SD	Range	Median	*p*-Value
CRT (μm)	336.8 ± 33.3	252–385	343.5	321.1 ± 24.6	266–392	323.5	*p* = 0.08
NFA (mm^2^)	4.20 ± 2.13	1.05–11.32	4.15	0.88 ± 0.60	0.12–2.51	0.71	*p* < 0.0001
VDSF (%)	44.73 ± 4.87	28.9–51.1	46.1	49.03 ± 3.62	37.4–55.0	49.5	*p* < 0.0001
VDD (%)	43.84 ± 4.54	37.0–53.0	44.55	51.10 ± 5.47	38.6–60.7	51.7	*p* < 0.0001
FAOR (mm^2^)	12.63 ± 4.28	4.07–20.87	12.9	14.85 ± 3.38	9.40–23.70	15.0	*p* = 0.05
FACC (mm^2^)	24.14 ± 1.44	21.9–26.6	24.2	25.3 ± 1.02	23.7–27.2	25.2	*p* < 0.001
FAZ (mm^2^)	0.28 ± 0.11	0.15–0.57	0.26	0.26 ± 0.09	0.05–0.41	0.27	*p* = 0.7
MD (dB)	5.0 ± 3.5	1.0–14.5	4.1	1.47–2.44	−1.8–7.2	0.7	*p* < 0.0001
BCVA	0.44 ± 0.29	0.06–0.9	0.4	0.90 ± 0.11	0.7–1.0	1.0	*p* < 0.0001

**Table 4:** Timepoint 3—measurement results in the study (*n* = 25) and control (*n* = 25) eyes and the statistical significance of the difference.

Variable	Study Eyes			Control Eyes			
	X ± SD	Range	Median	X ± SD	Range	Median	*p*-Value
CRT (μm)	330.0 ± 30.7	255–377	337.0	320.6 ± 25.4	267–389	321.0	*p* = 0.2
NFA (mm^2^)	3.67 ± 2.12	1.00–11.30	3.63	0.84 ± 0.61	0.15–2.53	0.62	*p* < 0.0001
VDSF (%)	44.90–4.89	28.0–50.9	46.10	48.85 ± 3.97	35.2–54.2	50.1	*p* < 0.0001
VDD (%)	44.7 ± 4.77	33.6–53.5	45.6	51.75 ± 5.67	37.6–60.8	52.5	*p* < 0.0001
FAOR (mm^2^)	13.64 ± 3.93	5.18–20.9	13.4	15.16 ± 3.40	9.27–22.95	15.9	*p* = 0.1
FACC (mm^2^)	24.26 ± 1.31	21.3–26.6	24.7	25.2 ± 1.21	23.0–27.5	25.5	*p* = 0.1
FAZ (mm^2^)	0.31 ± 0.14	0.15–0.73	0.29	0.26 ± 0.08	0.08–0.40	0.27	*p* = 0.3
MD (dB)	3.98 ± 2.99	0.1–10.6	3.65	0.91 ± 2.10	−1.8–7.1	0.20	*p* < 0.0001
BCVA	0.55 ± 0.27	0.1–1.0	0.6	0.92 ± 0.11	0.7–1.0	1.0	*p* < 0.0001

**Table 5:** Timepoint 4—measurement results in the study (*n* = 25) and control (*n* = 25) eyes and the statistical significance of the difference.

Variable	Study Eyes			Control Eyes			
	X ± SD	Range	Median	X ± SD	Range	Median	*p*-Value
CRT (μm)	317.2 ± 35.8	218–363	328.5	320.0 ± 24.0	271–384	320.4	*p* = 0.7
NFA (mm^2^)	3.33 ± 2.09	1.01–11.32	3.13	0.79 ± 0.54	0.15–2.08	0.53	*p* < 0.0001
VDSF (%)	45.09 ± 4.95	27.3–50.6	46.1	49.16 ± 3.75	36.1–53.9	50.3	*p* < 0.0001
VDD (%)	45.37 ± 4.70	35.1–53.6	455.2	51.5 ± 5.03	39.8–60.6	51.7	*p* < 0.0001
FAOR (mm^2^)	14.15 ± 3.95	6.0–21.2	14.1	15.17 ± 3.35	8.48–22.9	15.9	*p* = 0.3
FACC (mm^2^)	24.50 ± 1.23	21.0–26.5	24.8	25.19–1.11	23.1–27.4	25.3	*p* = 0.1
FAZ (mm^2^)	0.31 ± 0.11	0.17–0.67	0.29	0.27 ± 0.08	0.08–0.44	0.27	*p* = 0.2
MD (dB)	2.87–2.54	−0.7–8.2	2.45	0.57 ± 1.95	−2.0–6.4	0.00	*p* < 0.0001
BCVA	0.62 ± 0.26	0.1–1.0	0.6	0.94 ± 0.11	0.7–1.0	1.0	*p* < 0.0001

**Table 6:** Timepoint 5—measurement results in the study (*n* = 25) and control (*n* = 25) eyes and the statistical significance of the difference.

Variable	Study Eyes			Control Eyes			
	X ± SD	Range	Median	X ± SD	Range	Median	*p*-Value
CRT (μm)	316.6 ± 34.6	225–382	329.0	321.1 ± 22.4	276–387	322.5	*p* = 0.6
NFA (mm^2^)	3.14 ± 1.98	0.87–10.61	3.04	0.79 ± 0.55	0.13–2.08	0.53	*p* < 0.0001
VDSF (%)	45.22 ± 4.80	28.3–50.5	46.4	48.98 ± 3.52	36.5–54.7	50.2	*p* < 0.0001
VDD (%)	45.51 ± 4.66	35.3–53.9	45.5	51.42 ± 4.83	41.4–60.7	51.6	*p* < 0.0001
FAOR (mm^2^)	14.53 ± 3.79	6.1–21.1	14.4	15.75 ± 2.97	8.4–22.7	16.5	*p* = 0.2
FACC (mm^2^)	24.62 ± 1.09	21.5–26.5	24.9	24.84 ± 2.28	15.5–27.5	25.3	*p* = 0.1
FAZ (mm^2^)	0.31 ± 0.11	0.16–0.69	0.28	0.30 ± 0.14	0.09–0.87	0.28	*p* = 0.7
MD (dB)	2.11 ± 2.29	−0.8–7.6	1.4	0.26 ± 1.94	−2.6–6.2	−0.1	*p* < 0.001
BCVA	0.64 ± 0.28	0.1–1.0	0.7	0.94 ± 0.10	0.7–1.0	1.0	*p* < 0.0001

## Data Availability

Not applicable.
